# Structural Organization of Dibromodiazadienes in the Crystal and Identification of Br···O Halogen Bonding Involving the Nitro Group

**DOI:** 10.3390/molecules27165110

**Published:** 2022-08-11

**Authors:** Valentine G. Nenajdenko, Namiq G. Shikhaliyev, Abel M. Maharramov, Gulnar T. Atakishiyeva, Aytan A. Niyazova, Naila A. Mammadova, Alexander S. Novikov, Ivan V. Buslov, Victor N. Khrustalev, Alexander G. Tskhovrebov

**Affiliations:** 1 Department of Chemistry, M. V. Lomonosov Moscow State University, 1, Leninskie Gory, Moscow 119991, Russia; 2 Department of Organic Chemistry, Baku State University, Z. Xalilov 23, Baku AZ1000, Azerbaijan; 3 Department of Engineering and Applied Sciences, Azerbaijan State University of Economics, M.Mukhtarov 194, Baku AZ1001, Azerbaijan; 4 Institute of Chemistry, Saint Petersburg State University, Universitetskaya Nab. 7/9, Saint Petersburg 199034, Russia; 5 Research Institute of Chemistry, Peoples’ Friendship University of Russia, 6 Miklukho-Maklaya Street, Moscow 117198, Russia; 6 N.D. Zelinsky Institute of Organic Chemistry, Russian Academy of Sciences, 47 Leninsky Av., Moscow 119334, Russia

**Keywords:** non-covalent interactions, halogen bonding, azo dyes, DFT, QTAIM

## Abstract

Nitro functionalized dibromodiazadiene dyes were prepared and fully characterized including X-ray single crystal analysis. Electron deficient dibromodiazadienes were found to be able to act as donors of halogen bonding (XB), while the nitro group acted as an acceptor of the XB. Depending on the substituents, the Br···O XB competed with other weak interactions, and for some of the dyes, they even outcompeted the XB involving the nitro group. However, the nitro functionalized dibromoalkenes **6a** and **10a**, which had only the nitro moiety as the most plausible acceptor of the XB, reliably formed 1D chains via Br⋯O XB. Experimental work was supported by the DFT calculations and topological analysis of the electron density distribution within the framework of Bader’s theory (QTAIM method).

## 1. Introduction

Non-covalent interactions play a central role in many chemical phenomena, including catalysis, conformational changes, self-assembly in the solid state, molecular recognition etc. [1,2,3,4,5,6,7,8,9,10,11] The application of non-covalent interactions is among the most employed tools for the design of supramolecular materials. Hydrogen bonding (HB) is a ubiquitous interaction, which is often encountered in such artificial and natural systems. However, other weak interactions including halogen bonding (XB), have recently become the focus of researchers’ attention due to the similarity between the XB and HB; both interactions have a comparable strength, but XB shows a notable directionality [3,4,5,6,7,8,9,10,12,13,14,15,16].

**Scheme 1:**
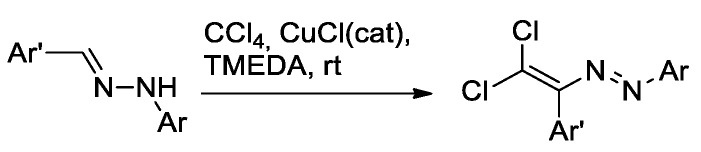
Reaction between aryl hydrazones and CCl_4_, induced by the copper catalyst.

Recently, Nenajdenko et al. discovered a remarkable carbon–carbon bond-forming reaction between aryl hydrazones and polyhaloalkanes, induced by the copper catalyst, and leading to halogenated diazabutadienes (Scheme 1) [17].

Furthermore, we demonstrated that the CCl_2_ moiety in easily polarizable dichlorodiazadienes can act as donors of XB [18,19]. We showed that the Hal···Hal interactions dictate a packing preference for halogenated dichlorodiazadienes, a newly discovered class of dyes.

In the course of our exploration of the novel Cu-catalyzed reaction between hydrazones and polyhaloalanes [18,19,20], and following our interest in non-covalent interactions [21,22,23], here we describe the coupling between CBr_4_ and nitro-functionalized hydrazones, which results in the formation of the mixture of dibromodiazadienes and dibromoalkenes via N_2_ extrusion. Multiple XB in the solid state for both dibromodiazadienes and dibromoalkenes were studied theoretically by means of DFT calculations and topological analysis of the electron density distribution within the formalism of Bader’s theory (QTAIM method).

## 2. Results and Discussion

**Scheme 2:**
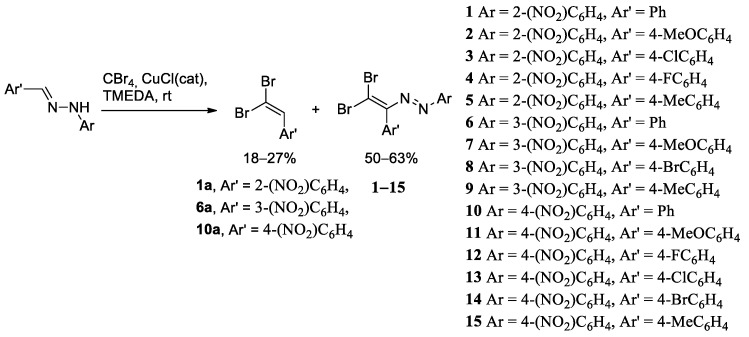
Copper-induced reaction between aryl hydrazones and CBr_4_.

Dibromodiazadienes 1–15 were prepared employing CBr_4_ (Scheme 2), in a similar fashion as earlier described dichlorodiazadienes [17,18]. Dibromo dyes were isolated in high yields (50–63%) as red solids. Interestingly, for the coupling of primary hydrazones with CBr_4_ we observed the formation of dibromoalkenes in a significant amount (19–27%, Scheme 2).

**Figure 1:**
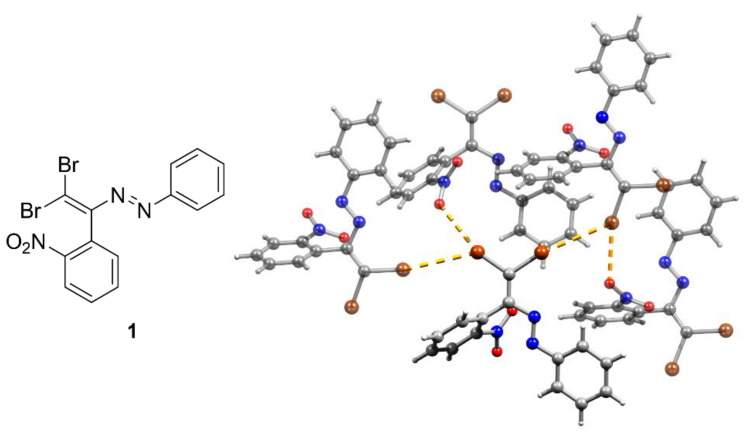
Ball-and-stick representation of **1** demonstrating Br···Br and Br···O XB in the crystal. Blue, brown, red, light gray and gray spheres represent nitrogen, bromine, oxygen, carbon and hydrogen atoms, respectively.

**Figure 2:**
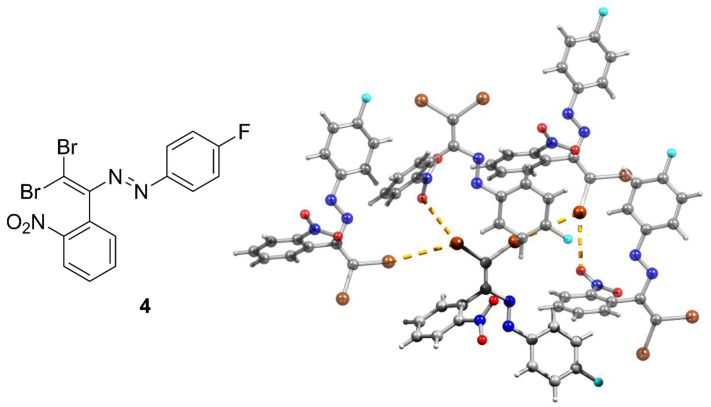
Ball-and-stick representation of **4** demonstrating Br···Br and Br···O XB in the crystal. Blue, brown, cyan, red, light gray and gray spheres represent nitrogen, bromine, fluorine, oxygen, carbon and hydrogen atoms, respectively.

**Figure 3:**
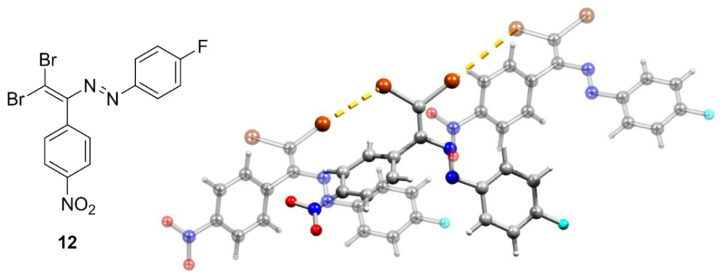
Ball-and-stick representation of **12** demonstrating Br···Br XB in the crystal. Blue, brown, cyan, red, light gray and gray spheres represent nitrogen, bromine, fluorine, oxygen, carbon and hydrogen atoms, respectively.

**Figure 4:**
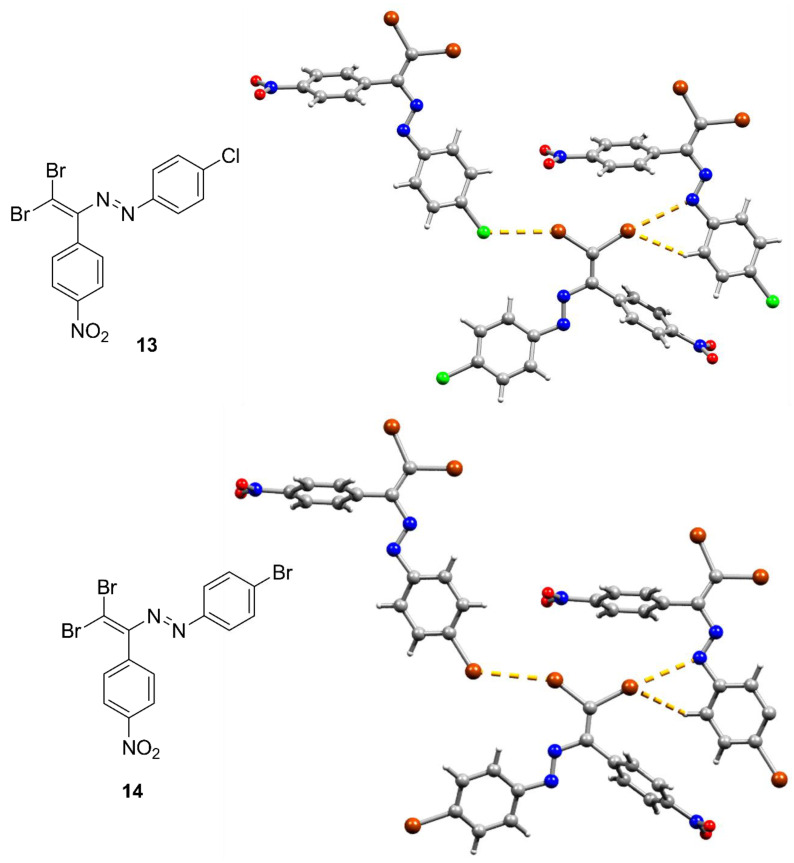
Ball-and-stick representation of **13** and **14** demonstrating Br···Cl, Br···Br and Br···N XB in the crystal. Blue, brown, green, red, light gray and gray spheres represent nitrogen, bromine, chlorine, oxygen, carbon and hydrogen atoms, respectively.

**Figure 5:**
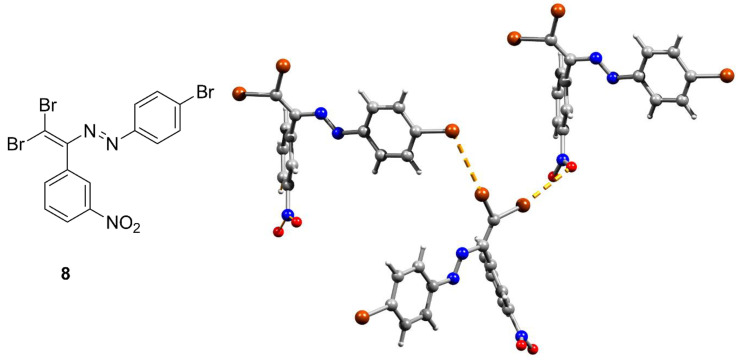
Ball-and-stick representations of **8** demonstrating Br···Br and Br···O XB in the crystal. Blue, brown, red, light gray and gray spheres represent nitrogen, bromine, oxygen, carbon, and hydrogen atoms, respectively.

**Figure 6:**
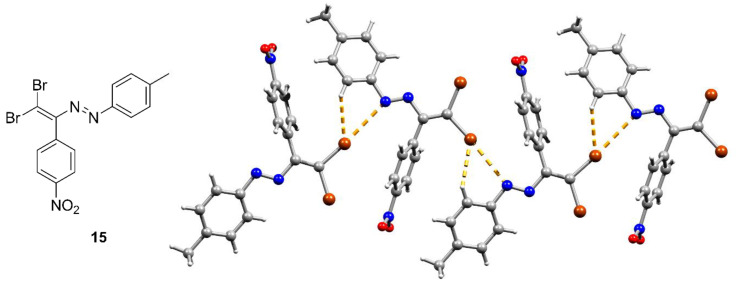
Ball-and-stick representations of **15** demonstrating “chelating” Br⋯N and Br⋯H interactions in the crystal. Blue, brown, red, light gray and gray spheres represent nitrogen, bromine, oxygen, carbon, and hydrogen atoms, respectively.

**Figure 7:**
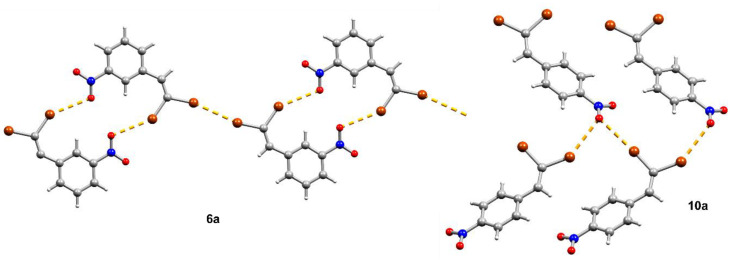
Ball-and-stick representations **6a** and **10a** demonstrating their self-assembly via Br···O XB in the crystal. Blue, brown, red, light gray and gray spheres represent nitrogen, bromine, oxygen, carbon, and hydrogen atoms, respectively.

The identity and purity of **1a**, **6a**, **10a** and **1–15** was confirmed by the ^1^H and ^13^C NMR spectroscopies and single crystal X-ray diffraction analysis for **1**, **8**, **13**, **15**, **6a** and **10a** (Figure 1, Figure 2, Figure 3, Figure 4, Figure 5, Figure 6 and Figure 7). Bond lengths and angles are similar to what was observed earlier for relevant diazabutadienes and azocompounds [17,19,24,25].

According to what we expected, the nitro group in the dibromo-dyes’ backbone acted as an acceptor of the XB involving C=CBr_2_ fragment and had a dramatic impact on the packing in the crystal. Compound 1, featuring *o*-nitrophenyl substituent by the C=C double bond, exhibited type 1 Br···Br contacts (Figure 1). Additionally, the nitro group formed Br···O XB with one of the bromine atoms of the C=CBr_2_ fragment (Figure 1).

Interestingly, the introduction of the fluorine in the para position of the aryl group by the azo functionality had no impact on the dyes self-assembly in the solid state: akin **1** compound **4** featured Br···Br and Br···O XB in the crystal, while the F atom was not involved in any XB (Figure 2). 

However, compound **12**, which is an isomer of **4** and contains a nitro group in a para position, did not exhibit Br···O XB (Figure 3). In this case, other weak interactions outcompeted the formation of the contact between the nitro group and XB donating Br atom. Like **1** and **4**, **12** also featured Br···Br XB, but they were rather type 2 contacts (Figure 3).

Interestingly, switching from the F to the Cl or Br substituents had a dramatic impact on the dyes’ self-assembly in the solid state. The neighboring dibromodiazadiene molecules in the crystal of **13** or **14** featured Br···Cl and Br···Br contacts, respectively, and a remarkable combination of “chelating” Br⋯N and Br⋯H non-covalent interactions (Figure 4). The latter type of supramolecular structural motif was not observed for the earlier described dichlorodiazadienes [18], and was arguably related to the larger size and softness of the Br atom in the dibromo dyes. No XB involving the nitro group was observed for **13** or **14**.

Furthermore, switching from the para (**14**) to the meta (**8**) nitro substitution had some interesting implications to the dyes’ self-assembly in the solid state. It was found that **8** also featured Br···Br contacts via one of the Br atoms of the CBr_2_ fragment. The second Br atom of the dibromoalkene fragment was involved in the Br⋯O XB with the nitro functionality (Figure 5). In this case, Br⋯O XB outcompeted the formation of “chelating” Br⋯N and Br⋯H non-covalent interactions.

Finally, when the Me group was in the para position of the aryl substituent by the azo fragment (compound **15**), only one of the Br atoms of the dibromodiazadiene was involved in the XB, “chelating” Br⋯N and Br⋯H interactions; the structural motif which was already found for **13** and **14** (Figure 6). No Br⋯O XB with the nitro functionality was observed for 15.

In addition, we obtained single crystals of dibromoalkenes **6a** and **10a** carrying the nitro group in the meta and para positions of the aryl substituent, respectively (Figure 7). An electron deficient dibromoalkene fragment was expectedly involved in the XB. In these cases, we expected that the only possible acceptor of the XB could be the nitro group, and it was indeed found to form the XB with the Br atoms (Figure 7).

**Figure 8:**
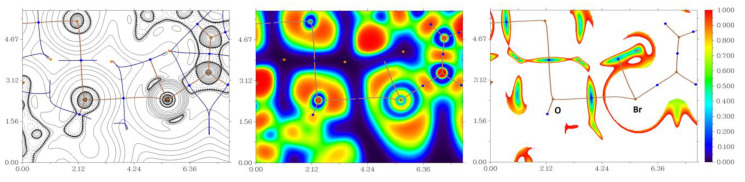
Contour line diagram of the Laplacian of electron density distribution ∇^2^ρ(**r**) displaying a corresponding number of alternating shells of charge concentration (solid lines) and charge depletion (thin and dashed lines), bond paths, and selected zero-flux surfaces (left), visualization of electron localization function (ELF, center) and reduced density gradient (RDG, right) analyses for intermolecular interactions of Br···NO_2_ in the X-ray structure **6a**. Bond critical points (3, −1) are shown in blue, nuclear critical points (3, −3) in pale brown, ring critical points (3, +1) in orange, bond paths are shown as pale brown lines, length units–Å, and the color scale for the ELF and RDG maps is presented in a.u.

**Figure 9:**
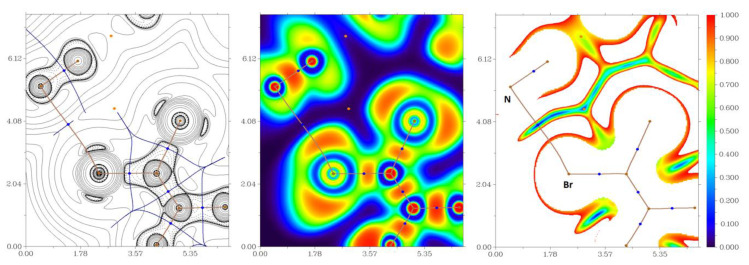
Contour line diagram of the Laplacian of electron density distribution ∇^2^ρ(**r**) displaying a corresponding number of alternating shells of charge concentration (solid lines) and charge depletion (thin and dashed lines), bond paths, and selected zero-flux surfaces (**left**), visualization of electron localization function (ELF, **center**) and reduced density gradient (RDG, **right**) analyses for intermolecular interactions of Br···NO_2_ in the X-ray structure **15**. Bond critical points (3, −1) are shown in blue, nuclear critical points (3, −3) in pale brown, ring critical points (3, +1) in orange, bond paths are shown as pale brown lines, length units–Å, and the color scale for the ELF and RDG maps is presented in a.u.

To prove the existence and approximately quantify the strength of intermolecular interactions of Br···NO_2_ in the obtained compounds, the DFT calculations followed by the topological analysis of the electron density distribution were carried out at the ωB97XD/6-311G* level of theory for model supramolecular associates (see Computational details and Table S1 in Supporting Information; note that inspection of the Cambridge Structural Database (CSD) reveals 10 examples of known X-ray structures featuring similar intermolecular interactions to Br···NO_2_, see Table S2). The existence of these non-covalent interactions was justified by the presence of bond critical points (3, −1) for appropriate intermolecular contacts and their lengths are shorter than the vdW radii sums of corresponding interacting atoms. Results of the QTAIM analysis are summarized in Table 1. The contour line diagrams of the Laplacian of electron density distribution ∇^2^ρ(**r**), bond paths, and selected zero-flux surfaces, visualization of electron localization function (ELF) and reduced density gradient (RDG) analyses for intermolecular interactions of Br···NO_2_ in the X-ray structures **6a** and **15** are shown in Figure 8 and Figure 9. 

## 3. Materials and Methods

General remarks: Unless stated otherwise, all the reagents used in this study were obtained from the commercial sources (Aldrich, TCI-Europe, Strem, ABCR). NMR spectra were recorded on a Bruker Avance 300 (^1^H: 300 MHz, Karlsruhe, Germany); chemical shifts (δ) are given in ppm relative to TMS, coupling constants (J) in Hz. Solvents were purified by distillation over the indicated drying agents and were transferred under Ar: Et_2_O (Mg/anthracene), CH_2_Cl_2_ (CaH_2_), hexane (Na/K). Flash chromatography: Merck Geduran^®^ Si 60 (Darmstadt, Germany) (40–63 μm). Compounds **4**, **12** and **14** were synthesized according to the literature [28,29,30].

Computational details: The single point calculations based on the experimental X-ray geometries have been carried out at the DFT level of theory using the dispersion-corrected hybrid functional ωB97XD [31] with the help of the Gaussian-09 [32] program package. The 6-311G* basis sets were used for all atoms. The topological analysis of the electron density distribution has been performed by using the Multiwfn program (version 3.7) [33]. The Cartesian atomic coordinates for model supramolecular associates are presented in Table S1, Supporting Information.

## 4. Synthetic part

### Synthesis of Dibromodiazadiens and Dibromoalkenes

A 20 mL screw neck vial was charged with DMSO (10 mL), phenylhydrazone (1 mmol), tetramethylethylenediamine (TMEDA) (295 mg, 2.5 mmol), CuCl (2 mg, 0.02 mmol) and CBr_4_ (1 mmol). After 1–3 hours (until TLC analysis showed complete consumption of corresponding Schiff base) the reaction mixture was poured into ~0.01 M solution of HCl (100 mL, ~pH = 2), and extracted with dichloromethane (3 × 20 mL). The combined organic phase was washed with water (3 ×50 mL), brine (30 mL), dried over anhydrous Na_2_SO_4_ and concentrated in vacuo. The residue was separated and purified by column chromatography on silica gel using appropriate mixtures of hexane and dichloromethane (3/1–1/1).


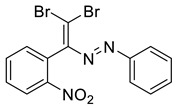


**1.** Red solid (63%), mp 144 °C. ^1^H NMR (300 MHz, Chloroform-*d*) δ 8.24 (d, *J* = 8.0 Hz, 1H, arom), 7.72 (d, *J* = 15.6 Hz, 3H, arom), 7.62 (t, *J* = 7.5 Hz, 1H, arom), 7.44 (d, *J* = 6.8 Hz, 3H, arom), 7.33 (d, *J* = 7.3 Hz, 1H, arom) ^13^C NMR (75 MHz, CDCl_3_) δ 154.4, 152.3, 133.7, 132.1, 132.0, 130.4, 130.1, 129.1, 124.5, 123.4, 109.9.


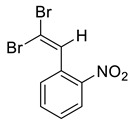


**1a**. Colorless solid (25%), mp 58 °C. ^1^H NMR (300 MHz, Chloroform-*d*) δ 8.15 (d, *J* = 8.2 Hz, 1H), 7.80 (s, ^1^H), 7.72–7.67 (m, 1H), 7.64–7.54 (m, 2H). ^13^C NMR (75 MHz, CDCl_3_) δ 129.4, 128.9, 127.0, 124.8, 120.2, 119.0, 88.6.


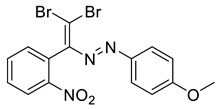


**2**. Red solid (57%), mp 122 °C. ^1^H NMR (300 MHz, Chloroform-*d*) δ 8.21 (dd, *J* = 8.1, 1.0 Hz, 1H), 7.75–7.67 (m, 3H), 7.64–7.57 (m, 1H), 7.32 (dd, *J* = 7.5, 1.4 Hz, 1H), 6.91 (d, *J* = 9.0 Hz, 2H), 3.85 (s, 3H). ^13^C NMR (75 MHz, CDCl_3_) δ 162.9, 154.2, 147.8, 146.8, 133.6, 132.1, 130.7, 129.9, 125.5, 124.4, 114.2, 107.4, 55.6. 1a (24%).


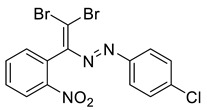


**3**. Red solid (63%), mp 114 °C. ^1^H NMR (300 MHz, DMSO-*d*_6_) δ 8.25 (d, *J* = 8.1 Hz, ^1^H), 7.87 (d, *J* = 7.4 Hz, 1H), 7.77 (t, *J* = 7.7 Hz, 1H), 7.66–7.53 (m, 5H). ^13^C NMR (75 MHz, DMSO) δ 154.3, 150.7, 147.7, 137.4, 135.1, 132.8, 131.4, 130.3, 129.5, 125.0, 124.8, 113.3. 1a (18%).


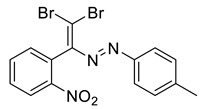


**5**. Red solid (54%), mp 108 °C. ^1^H NMR (300 MHz, Chloroform-*d*) δ 8.22 (d, *J* = 9.2 Hz, 1H), 7.74–7.57 (m, 4H), 7.32 (d, *J* = 7.5 Hz, 1H), 7.22 (d, *J* = 8.2 Hz, 2H), 2.38 (s, 3H). ^13^C NMR (75 MHz, CDCl_3_) δ 154.4, 150.5, 147.7, 142.9, 133.7, 132.1, 130.5, 130.0, 129.8, 124.5, 123.5, 108.9, 21.7. 1a (22%).


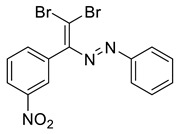


**6**. Red solid (50%), mp 145 °C. ^1^H NMR (300 MHz, DMSO-*d*_6_) δ 8.25 (d, *J* = 8.1 Hz, 1H), 7.88 (t, *J* = 7.4 Hz, 1H), 7.77 (t, *J* = 7.7 Hz, 1H), 7.69–7.50 (m, 6H). ^13^C NMR (75 MHz, DMSO) δ 154.3, 150.7, 147.7, 137.4, 135.1, 132.8, 131.4, 130.3, 129.5, 128.0, 124.8, 113.3.


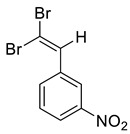


**6a**. yellow solid (27%), mp 50 °C. ^1^H NMR (300 MHz, Chloroform-*d*) δ 8.46 (s, 1H), 8.22 (d, *J* = 8.0 Hz, 1H), 7.85 (d, *J* = 7.8 Hz, 1H), 7.65–7.53 (m, 2H). ^13^C NMR (75 MHz, CDCl_3_) δ 140.1, 136.8, 134.4, 134.2, 129.4, 126.7, 123.1, 93.3.


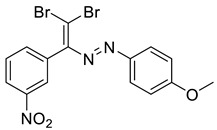


**7**. Red solid (56%), mp 140 °C. ^1^H NMR (300 MHz, Chloroform-*d*) δ 8.28 (d, *J* = 8.2 Hz, 1H), 8.06 (s, 1H), 7.77 (d, *J* = 9.0 Hz, 2H), 7.62 (t, *J* = 7.9 Hz, 1H), 7.50 (d, *J* = 7.6 Hz, 1H), 6.95 (d, *J* = 9.0 Hz, 2H), 3.88 (s, 3H). ^13^C NMR (75 MHz, CDCl_3_) δ 163.0, 154.1, 148.0, 147.0, 136.4, 136.1, 129.1, 125.5, 125.1, 123.5, 114.4, 108.9, 55.6. 6a (23%).


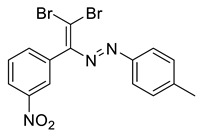


**9**. Red solid (61%), mp 135 °C. ^1^H NMR (300 MHz, Chloroform-*d*) δ 8.32–8.26 (m, 1H), 8.11–8.05 (m, 1H), 7.70 (d, *J* = 8.3 Hz, 2H), 7.62 (t, *J* = 7.9 Hz, 1H), 7.50 (dt, *J* = 7.6, 1.2 Hz, 1H), 7.26 (d, *J* = 8.1 Hz, 2H), 2.40 (s, 3H). ^13^C NMR (75 MHz, CDCl_3_) δ 154.2, 150.7, 148.0, 143.1, 136.2, 136.1, 129.9, 129.2, 125.1, 123.5, 123.5, 110.5, 21.7. 6a (21%).


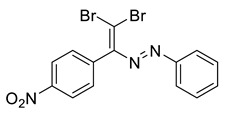


**10**. Red solid (63%), mp 118 °C. ^1^H NMR (300 MHz, Chloroform-*d*) δ 8.35–8.24 (m, 2H), 7.80 (dd, *J* = 8.0, 1.7 Hz, 2H), 7.54–7.43 (m, 3H), 7.39–7.33 (m, 2H). ^13^C NMR (75 MHz, CDCl_3_) δ ^13^C NMR (75 MHz, CDCl_3_) δ 154.6, 152.5, 147.8, 141.4, 132.3, 131.0, 129.3, 123.7, 123.4, 111.2. 


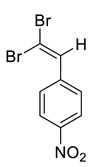


**10a**. Orange solid (19%), mp 55 °C. ^1^H NMR (300 MHz, Chloroform-*d*) δ 8.15 (d, *J* = 8.2 Hz, 1H, arom), 7.80 (s, 1H, =CH), 7.68 (d, *J* = 7.8 Hz, 1H, arom), 7.64–7.52 (m, 2H, arom). ^13^C NMR (75 MHz, CDCl_3_) δ 129.4, 128.9, 127.0, 124.8, 120.2, 88.6.


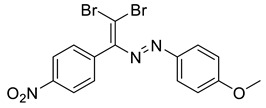


**11.** Red solid (62%), mp 133 °C. ^1^H NMR (300 MHz, Chloroform-*d*) δ 8.28 (d, *J* = 8.8 Hz, 2H), 7.77 (d, *J* = 9.0 Hz, 2H), 7.35 (d, *J* = 8.8 Hz, 2H), 6.94 (d, *J* = 9.0 Hz, 2H), 3.87 (s, 3H). ^13^C NMR (75 MHz, CDCl_3_) δ 163.1, 154.5, 147.7, 147.1, 141.8, 131.2, 131.0, 125.5, 123.3, 114.4, 108.4, 55.6. 10a (18%).


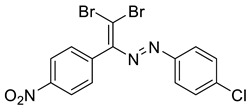


**13**. Red solid (58%), mp 170 °C. ^1^H NMR (300 MHz, Chloroform-*d*) δ 8.37–8.28 (m, 2H), 7.76–7.68 (m, 2H), 7.45–7.40 (m, 2H), 7.39–7.32 (m, 2H). ^13^C NMR (75 MHz, CDCl_3_) δ 154.6, 150.9, 147.9, 141.2, 138.3, 130.9, 129.5, 124.6, 123.4, 111.9. 10a (24%).


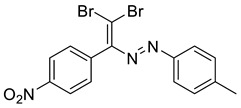


**15.** Red solid (61%), mp 122 °C. ^1^H NMR (300 MHz, Chloroform-*d*) δ 8.38–8.22 (m, 2H), 7.74–7.65 (m, 2H), 7.36 (dq, *J* = 9.1, 2.2 Hz, 2H), 7.25 (d, *J* = 8.1 Hz, 2H), 2.19 (s, 3H). ^13^C NMR (75 MHz, CDCl_3_) δ 154.8, 150.7, 143.1, 140.5, 140.1, 130.9, 129.9, 128.0, 123.4, 123.3, 21.6. 10a (25%).

## 5. Conclusions

In summary, here we report the synthesis and structural characterization of 11 dibromodiazadiene dyes carrying the nitro group in the backbone. An electron deficient and easily polarizable dibromodiazadiene fragment was involved in multiple XB interactions involving the Br atoms, the strength of which are comparable with energies of Br···Br noncovalent interactions in crystals of Sn(IV) (2.1-4.3 kcal/mol) [14], Bi(III) (1.4–2.5 kcal/mol) [17], and Au(III) (1.6 kcal/mol) [20] bromide complexes, Br···N halogen bonds in 2,5-dibromothiophenes (2.5-2.9 kcal/mol) [15], Br···O contacts in [{AgL}_2_Mo_8_O_26_]^2−^ complexes (2.1 kcal/mol) [19], and Cl···Br halogen bonding in bromoaryl-substituted dichlorodiazabutadienes (1.2–1.8 kcal/mol) [13]. For some of the dyes, “chelating” Br⋯N and Br⋯H interactions were identified, which were not observed dichlorodiazadienes. The nitro group was involved in the XB for some cases; however, for some dyes, other weak interactions outcompeted the Br⋯O XB formation. In contrast, the nitro decorated dibromoalkenes **6a** and **10a**, which had only the nitro moiety as the most plausible acceptor of the XB, reliably formed 1D chains via Br⋯O XB. Experimental work was supported by the DFT calculations and topological analysis of the electron density distribution within the framework of Bader’s theory (QTAIM method). 

## Figures and Tables

**Table 1:** Values of the density of all electrons–ρ(**r**), Laplacian of electron density–∇^2^ρ(**r**) and appropriate λ_2_ eigenvalue, energy density–H_b_, potential energy density–V(**r**), and Lagrangian kinetic energy–G(**r**) (a.u.) at the bond critical point (3, −1), corresponding to intermolecular interactions Br···NO_2_ in the obtained X-ray structures and estimated strength for these interactions E_int_ (kcal/mol).

Contact *	ρ(r)	∇^2^ρ(r)	λ_2_	H_b_	V(r)	G(r)	E_int_ **
**1**							
Br···O 2.944 Å	0.012	0.048	−0.012	0.001	−0.009	0.010	3.6
**4**							
Br···O 2.983 Å	0.012	0.045	−0.012	0.002	−0.008	0.010	3.6
**6a**							
Br···O 3.134 Å	0.010	0.036	−0.010	0.001	−0.007	0.008	2.9
**10**							
Br···O 3.137 Å	0.009	0.033	−0.009	0.001	−0.006	0.007	2.5
**10a**							
Br···O 3.128 Å	0.010	0.035	−0.010	0.002	−0.006	0.008	2.9
Br···O 3.155 Å	0.010	0.034	−0.010	0.001	−0.006	0.007	2.5
Br···O 3.143 Å	0.009	0.034	−0.009	0.001	−0.006	0.007	2.5
Br···O 3.163 Å	0.009	0.033	−0.009	0.001	−0.006	0.007	2.5
**14**							
Br···N 3.438 Å	0.006	0.022	−0.006	0.002	−0.003	0.004	1.4
Br···N 3.419 Å	0.006	0.023	−0.006	0.002	−0.003	0.005	1.8
**15**							
Br···N 3.411 Å	0.006	0.023	−0.006	0.002	−0.003	0.005	1.8

* The Bondi’s van der Waals radii for Br, O, and N atoms are 1.83, 1.52, and 1.55 Å, respectively [26]. ** E_int_ = 0.57G(**r**) (correlation developed specifically for noncovalent interactions involving bromine atoms) [27].

## Supplementary Materials

The following are available online at https://www.mdpi.com/article/10.3390/molecules27165110/s1, Crystal structure determinations, Table S1: Crystal data and structure refinement for **1**, **6a**, **8**, **10a**, **13** and **15**. Table S2: Known X-ray structures featuring intermolecular interactions Br···NO2 from the Cambridge Structural Database (CSD) [34,35,36,37,38,39,40,41].
