# Supplementary material for: Structural Organization of Dibromodiazadienes in the Crystal and Identification of Br···O Halogen Bonding Involving the Nitro Group
Source: Molecules. 2022 Aug 11;27(16):5110. doi: 10.3390/molecules27165110 (PMC9414975; doi:10.3390/molecules27165110)
Supplement: Supplementary file 1 [file molecules-27-05110-s001.zip › molecules-1843506-supplementary.pdf]

Crystal structure determinations

The single-crystal X-ray diffraction data for **1**, **6a**, **8** and **10a** were collected on a three-circle Bruker D8 QUEST PHOTON-III CCD diffractometer (T = 100 K, λ(Mo$K\alpha$) = 0.71073 Å, graphite monochromator, ω and φ scanning mode). The data were indexed and integrated using the *SAINT* program [S1], and then scaled and corrected for absorption using the *SADABS* program [S2]. The single-crystal X-ray diffraction data for **13** and **15** were collected at the 'RSA' beamline (T = 100 K, λ = 0.79475 Å) of the Kurchatov Synchrotron Radiation Source. In total, 720 frames were collected with an oscillation range of 1.0° in the φ scanning mode using two different orientations for each crystal. The semi-empirical correction for absorption was applied using the *Scala* program [S3]. The data were indexed and integrated using the utility *iMOSFLM* from the CCP4 software suite [S4, S5]. For details, see **Table S1**.

The structures were solved by intrinsic phasing modification of direct methods [S6] and refined by a full-matrix least-squares technique on F^2 with anisotropic displacement parameters for all non-hydrogen atoms. The hydrogen atoms were placed in calculated positions and refined within the riding model with fixed isotropic displacement parameters [U_{iso}(H) = 1.5U_{eq}(C) for the methyl groups and 1.2U_{eq}(C) for the other groups]. All calculations were carried out using the *SHELXTL* program [S7, S8].

Crystallographic data for **1**, **6a**, **8**, **10a**, **13** and **15** have been deposited with the Cambridge Crystallographic Data Center, CCDC 2189773 (**1**), CCDC 2189774 (**6a**), CCDC 2189348 (**8**), CCDC 2189775 (**10a**), CCDC 2189776 (**13**), CCDC 2189777 (**15**). Copies of this information may be obtained free of charge from the Director, CCDC, 12 Union Road, Cambridge CB2 1EZ, UK (fax: +44 1223 336033; e-mail: deposit@ccdc.cam.ac.uk or www.ccdc.cam.ac.uk).

References

[S1] Bruker, *SAINT*, *V8.37A*, Bruker AXS Inc., Madison, WI, **2018**.

[S2] L. Krause, R. Herbst-Irmer, G.M. Sheldrick, D. Stalke, *J. Appl. Cryst*. **2015**, *48*, 3-10.

[S3] Evans, P. *Acta Crystallogr*. **2006**, D62, 72-82.

[S4] Battye, T.G.G.; Kontogiannis, L.; Johnson, O.; Powell, H.R.; Leslie, A.G.W. *Acta Crystallogr*. **2011**, D67, 271-281.

[S5] Winn, M.D.; Ballard, C.C.; Cowtan, K.D.; Dodson, E.J.; Emsley, P.; Evans, P.R.; Keegan, R.M.; Krissinel, E.B.; Leslie, A.G.W.; McCoy, A.; McNicholas, S.J.; Murshudov, G.N.; Pannu, N.S.; Potterton, E.A.; Powell, H.R.; Read, R.J.; Vagin, A.; Wilson, K.S. *Acta Crystallogr*. **2011**, D67, 235-242.

[S6] Sheldrick, G.M. *Acta Crystallogr*. **2015**, *A71*, 3-8.

[S7] Sheldrick, G.M. *Acta Crystallogr*. **2008**, *A64*, 112-122.

[S8] Sheldrick, G.M. *Acta Crystallogr*. **2015**, *C71*, 3-8.

Table S1. Crystal data and structure refinement for **1**, **6a**, **8**, **10a**, **13** and **15**.

Identification code	**1**	**6a**	**8**	**10a**	**13**	**15**
Empirical formula	$C_{14}H_9N_3O_2Br_2$	$C_8H_5NO_2Br_2$	$C_{14}H_8N_3O_2Br_3$	$C_8H_5NO_2Br_2$	$C_{14}H_8N_3O_2ClBr_2$	$C_{15}H_{11}N_3O_2Br_2$
Formula weight	411.06	306.95	489.96	306.95	445.48	425.07
Crystal size, mm^3	0.17 × 0.21 × 0.35	0.10 × 0.13 × 0.53	0.05 × 0.06 × 0.34	0.09 × 0.33 × 0.59	0.10 × 0.10 × 0.20	0.10 × 0.12 × 0.15
Crystal system	Orthorhombic	Monoclinic	Orthorhombic	Monoclinic	Orthorhombic	Orthorhombic
Space group	*P*bcn	$P2_1/c$	*P*bca	*P*n	$Pna2_1$	$Pna2_1$
a, Å	14.9390(3)	3.92910(10)	6.6579(1)	3.8523(1)	13.853(3)	13.843(3)
b, Å	15.0262(3)	16.1045(6)	15.7683(3)	7.1425(2)	13.474(3)	13.558(3)
c, Å	13.1009(2)	14.4329(5)	29.0301(6)	16.4527(4)	8.2790(6)	8.2810(17)
α, deg.	90	90	90	90	90	90
β, deg.	90	96.1786(11)	90	96.5953(7)	90	90
γ, deg.	90	90	90	90	90	90
V, Å^3	2940.84(9)	907.95(5)	3047.69(10)	449.70(2)	1545.3(5)	1554.2(6)
Z	8	4	8	2	4	4
Density (calc.), g/cm^3	1.857	2.245	2.136	2.267	1.915	1.817
Absorption coefficient, mm^{-1}	5.520	8.890	7.951	8.975	7.151	6.876
F(000)	1600	584	1872	292	864	832
θ range, deg.	2.47–34.37	2.53–33.16	2.58–32.58	2.49–35.64	2.36–30.97	2.35–30.98
Index ranges	$-23 \le h \le 23$<br>$-23 \le k \le 23$<br>$-20 \le l \le 20$	$-6 \le h \le 6$<br>$-24 \le k \le 24$<br>$-22 \le l \le 22$	$-10 \le h \le 10$<br>$-23 \le k \le 23$<br>$-43 \le l \le 43$	$-6 \le h \le 6$<br>$-11 \le k \le 11$<br>$-26 \le l \le 26$	$-17 \le h \le 17$<br>$-17 \le k \le 17$<br>$-10 \le l \le 9$	$-17 \le h \le 17$<br>$-17 \le k \le 17$<br>$-10 \le l \le 10$
Reflections collected	95218	7559	67179	30661	18617	19560
Independent reflections, R_{int}	6171, 0.041	3449, 0.028	5538, 0.033	4167, 0.036	3309, 0.065	3523, 0.046
Reflections observed ($I>2\sigma(I)$)	5247	3094	4620	4101	3192	3223
Final R_1/wR_2 indices, $I>2\sigma(I)$	0.024 / 0.062	0.032 / 0.071	0.024 / 0.060	0.026 / 0.065	0.036 / 0.088	0.042 / 0.106
Final R_1/wR_2 indices (all data)	0.033 / 0.066	0.043 / 0.076	0.034 / 0.064	0.026 / 0.066	0.037 / 0.089	0.046 / 0.109
Goodness-of-fit on F^2	1.051	1.050	1.047	1.050	1.031	1.037
T_{min}, T_{max}	0.043, 0.092	0.028, 0.104	0.019, 0.058	0.010, 0.059	0.280, 0.480	0.370, 0.490
Extinction coefficient	–	–	–	–	0.0089(9)	0.0036(3)
$\Delta\rho_{max}$ / $\Delta\rho_{min}$, e·Å^{-3}	0.696 / -0.693	0.460 / -0.569	0.675 / -0.518	0.478 / -0.639	0.545 / -0.778	1.509 / -0.845

Table S2. Known X-ray structures featuring intermolecular interactions Br···NO_2 from the Cambridge Structural Database (CSD).

CSD Refcode	Length of Br···NO_2 contacts	Reference
AJAREI	Br···O: 2.658 and 2.779 Å	M. Yoshizawa, M. Nagao, K. Umemoto, K. Biradha, M. Fujita, S. Sakamoto, K. Yamaguchi (2003) Chem. Commun., 1808.
DAFVEO	Br···O: 3.152 and 3.177 Å	A. A. Tehrani, S. Abedi, A. Morsali (2017) Cryst. Growth Des., 17, 255.
DARXEC	Br···O: 3.122 Å	S. Maiti, T. K. Achar, P. Mal (2017) Org. Lett., 19, 2006.
MIFKOE	Br···O: 3.116 and 3.218 Å	D. E. Williams, C. R. Martin, E. A. Dolgopolova, A. Swifton, D. C. Godfrey, O. A. Ejegbavwo, P. J. Pellechia, M. D. Smith, N. B. Shustova (2018) J. Am. Chem. Soc., 140, 7611.
MIFROJ	Br···N: 3.377 Å	Yu. M. Chumakov, V. I. Tsapkov, G. Bocelli, M. Neuburger, A. P. Gulya (2006) Koord. Khim., 32, 775.
MIQHUR	Br···O: 3.253 Å	J. Jiang, P. Vairaprakash, K. R. Reddy, T. Sahin, M. Phani Pavan, E. Lubian, J. S. Lindsey (2014) Org. Biomol. Chem., 12, 86.
NOJTIS	Br···O: 3.030 and 3.071 Å	R. Puttreddy, A. Peuronen, M. Lahtinen, K. Rissanen (2019) Cryst. Growth Des., 19, 3815.
RURTIJ	Br···N: 3.317 Å	R. Raja, S. Kandhasamy, P. T. Perumal, A. Subbiah Pandi (2015) Acta Crystallogr., Sect. E: Cryst.Commun., 71, o648.

SOYGIX	Br···O: 3.348 Å and Br···N: 3.294 Å	A. Salhin, N. Abdul Razak, I. A. Rahman (2009) Acta Crystallogr., Sect. E: Struct. Rep. Online, 65, o1221.
WAKNON	Br···O: 3.299, 3.015, and 3.084 Å	A. de Bettencourt-Dias, P. S. Barber, S. Viswanathan, D.T. de Lill, A. Rollett, G. Ling, S. Altun (2010) Inorg. Chem., 49, 8848.
